# Interaction learning control with movement primitives for lower limb exoskeleton

**DOI:** 10.3389/fnbot.2022.1086578

**Published:** 2022-12-20

**Authors:** Jiaqi Wang, Dongmei Wu, Yongzhuo Gao, Wei Dong

**Affiliations:** State Key Laboratory of Robotics and Systems, Harbin Institute of Technology, Harbin, China

**Keywords:** human–robot interaction, hierarchical control, lower limb exoskeleton, reinforcement learning, movement primitives

## Abstract

Research on robotic exoskeletons both in the military and medical fields has rapidly expanded over the previous decade. As a human–robot interaction system, it is a challenge to develop an assistive strategy that makes the exoskeleton supply efficient and natural assistance following the user's intention. This paper proposed a novel interaction learning control strategy for the lower extremity exoskeleton. A powerful representative tool probabilistic movement primitives (ProMPs) is adopted to model the motion and generate the desired trajectory in real-time. To adjust the trajectory by the user's real-time intention, a compensation term based on human–robot interaction force is designed and merged into the ProMPs model. Then, compliant impedance control is adopted as a low-level control where the desired trajectory is put into. Moreover, the model will be dynamically adapted online by penalizing both the interaction force and trajectory mismatch, with all the parameters that can be further learned by learning algorithm PI^BB^. The experimental results verified the effectiveness of the proposed control framework.

## Introduction

Technological improvements have led to the prosperous development of lower extremity exoskeletons for the physical assistance and recovery of human locomotion since the 1960s (Mosher, 1967). Major gains in robotic hardware, electronics, actuators, sensors and energy supplies have propelled the use and acceptance of viable prototypes further. A significant issue that still remains is how to effectively control the exoskeletons to maximize the benefits of these robotic devices. Unlike with other technologies, there is not a general convergence of solutions for exoskeleton control as a very wide variety of controls are used (Young and Ferris, 2016). The intended use and the target individuals vary, as well as the development of a single control strategy for each particular design. Therefore, the control strategies should be considered from the actual application requirements.

For the exoskeletons that are used for walking assistance in daily living for able-bodied and elderly individuals, which is the focus of this paper, the aim of control is not only to provide appropriate assistance but also to make the robots actively cooperate with the human user. In this case, it is important for the exoskeleton to possess the cognition of human movement and encode it intelligently in order to achieve flexible and coordinated human-robot cooperation.

Human movement modeling has been extensively investigated by researchers in the field of bipedal walking. The most classic strategy that is based on dynamic model and stability criteria, like the link model (Hirai et al., 1998; Fu and Chen, 2008), inverted pendulum model (Yokoi et al., 2001; Komura et al., 2005; Kazemi and Ozgoli, 2019), zero-moment point (Kagami et al., 2002; Vukobratović and Borovac, 2004; Huang and Nakamura, 2005; Al-Shuka et al., 2016; He et al., 2017) have been widely used. This kind of method has over-reliance on the model, mostly expensive computation, and poor adaptability to the environment (Kazemi and Ozgoli, 2019). Besides, the exoskeletons are wearable and literally work in parallel with humans which leads to higher requirements for flexibility. In order to encode and reproduce human motion rather than just copy, the approaches learning from the demonstration have gained considerable interest in robot systems (Deng et al., 2018; Yang et al., 2018).

In our case, lower limb exoskeletons can possess a better understanding of human behavior and reproduce it by learning human movements. Movement primitives (MPs) is a well-established approach to modular robot movement (Schaal et al., 2003; Schaal, 2006; Kulić et al., 2012). Dynamic movement primitives (DMPs) presented by Ijspeert et al. (2002, 2013) has been widely used in exoskeleton systems (Huang et al., 2018; Yang et al., 2018). In Huang et al. (2018), motion trajectories are modeled with DMPs and learned with locally weighted regression method. Except for a powerful representative model, it is also necessary that the model should be adjustable online, for the benefit of the different users (Tran et al., 2014), and to reduce the effect of uncertainties and disturbances. The exoskeleton is required to continuously improve the trajectory generation by optimizing the objective function. Reinforcement learning (RL) (Schmidhuber, 2015) is one of the most general frameworks of learning control to provide truly self-autonomous learning systems. PI^2^ (Theodorou et al., 2010) is a reinforcement learning policy improvement algorithm that combines optimal control and dynamic programming. Lots of works have illustrated the functionality of PI^2^ in a complex robot learning scenario (Tran et al., 2014; Huang et al., 2018), it offers currently one of the most efficient, numerically robust, and easy to implement algorithms for RL. Yuan et al. (2019) proposed a trajectory-learning scheme based on PI^2^ combined with DMP for motion generation.

The combination of DMPs and PI^2^ performs well, but there's still room for improvement. DMPs has some limitations, like the generalization to new situations (Paraschos et al., 2018). To this effect, a novel ProMPs is proposed by Paraschos et al. (2013, 2018), which incorporates a variety of advantages from other well-known previous MP representations (d'Avella and Bizzi, 2005; Schaal et al., 2005; Kober et al., 2010). As for PI^2^, the exploration and parameter update methods are slightly complicated for an online system. Stulp and Sigaud (2012) proposed a new algorithm PI^BB^, which is a simplified version of PI^2^ but has better performance. PI^BB^ belongs to black-box optimization in essence, and it has been proven that PI^BB^ outperform PI^2^ in terms of convergence speed and final cost. Therefore, in our previous work, ProMPs combined with PI^BB^ are innovatively adopted to model the motion in lower limb exoskeletons, and the effectiveness has been verified under zero-mode control that the motion generation process is more quickly and accurately.

In this paper, we present an interaction learning control strategy for the lower limb exoskeleton, which is based on previous work motion generation research. The motion learning part is still based on the powerful representative tool ProMPs to generate desired trajectories. For considering the current intention of the user, the human–robot interaction (HRI) shouldn't be ignored in assistive mode control, which is an important indicator of the naturalness and comfort of the exoskeleton HRI system. Therefore, we integrate the real-time HRI force into motion online generation. In specific, a compensation term modeled by HRI force, which can reflect the user's current intention, is designed and incorporated in ProMPs. Also, the learning algorithm PI^BB^ is adopted to tune the parameters of the whole new model for different gait patterns. In this way, the exoskeleton will not only have a better understanding and reproduction of human motion, but also can quickly respond to the new situation. And based on that, to complete the entire hierarchical control for assistive mode, then the very efficient and often adopted method, impedance control, is used in low-level control, endowing compliance between the exoskeleton actuators and the user (Hogan, 1984). Experimental results reveal that our method can model the present motion more precisely and quicken the convergence of HRI force. The performance of the method meets the practical requirements in the application of the lower limb assistant exoskeleton.

The structure of this paper is organized as follows. Section Methods introduces the process and the details of the proposed interactive motion learning strategy. In Section Experiments and analyses, the experiments are carried out, and the results are presented and analyzed. Finally, the paper is concluded in Section Conclusion and future work.

## Methods

**Figure 1:**
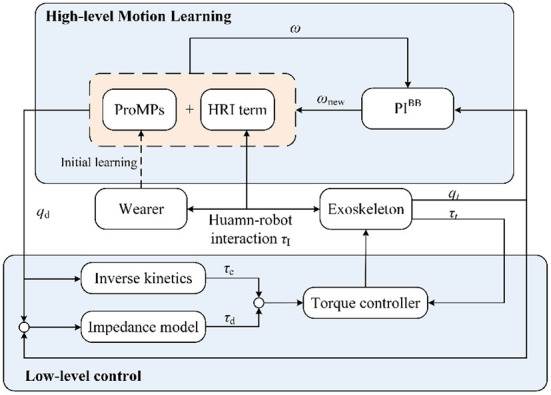
The interaction learning control framework.

This section presents the methodology details of the proposed interaction learning control strategy. Figure 1 illustrates the whole framework, which can be seen as a hierarchical control. In high-level motion learning, the trajectory generation ProMPs model is firstly built in offline initial modeling. Besides, the model is incorporated with a compensation term, which is a transformation of HRI force that can reflect the user's current intention. In the actual application, the real-time position will be put into PI^BB^ and optimize the model further. Afterward, the low-level control works on a new generated trajectory. The following Motion generation model, Motion model compensation, and Motion model adaptive learning section introduces motion learning sequential, and the Section Low-level control is the low-level control part. The meaning of the letters in the figure will be introduced in the corresponding part.

### Motion generation model

The fundamental of the motion learning part is ProMPs model. The concept of ProMPs introduced in d'Avella and Bizzi (2005) is a probability distribution over robot trajectories. To establish ProMPs motion model, the first is the representation of the probabilistic trajectory. A joint of the limb of the exoskeleton corresponds to a degree of freedom. To facilitate the description of the robot's motion trajectory distribution, *q*_*t*_ and${\dot{q}}_{t}$are used to respectively represent the joint angular position and joint angular velocity of each degree of freedom at time *t*. A movement trajectory of time length *T* is modeled by $y_{t}={\left\{q_{t}\right\}}_{t=1}^{T}$. For encoding the time-varying variance of movements, ω is used to compactly represent a trajectory as an underlying weight vector. The trajectory is given as a linear basis function model,


(1) $$\begin{array}{l} y_{t}=\left[\begin{array}{c} q_{t}\\ {\dot{q}}_{t} \end{array}\right]=\Phi_{t}^{T}\omega +\varepsilon_{y} \end{array}$$

where $\Phi_{t}^{\text{‏}}=\left[\phi_{t},{\dot{\phi }}_{t}\right]$ is the *N* × 2 dimensional time-dependent basis matrix, *N* defines the number of basis functions of each degree of freedom. $\varepsilon_{y}\sim N\left(0,\underset{y}{\sum }\right)$ Gaussian noise with 0 mean.

Temporal modulation is needed for modeling the human walking motion because the speed of walking is not fixed. A phase variable *z* is introduced to separate the movement from the time signal. The phase can be any function that monotonically increases with time *z*_*t*_, and the speed of the movement can be modulated by modifying the rate of the phase variable. In this paper, *z*_*t*_ is adopted as equation (5),


(2) $$\begin{array}{l} z_{t}=\alpha t. \end{array}$$

At the beginning of the gait movement, phase *z*_0_ is defined as 0 and *z*_E_ = 1 at the end. The basis function φ_*t*_ now directly depends on the phase instead of time,


(3) $$\begin{array}{l} \phi_{t}=\phi \left(z_{t}\right). \end{array}$$

The choice of the basis functions depends on the type of movement. For human walking motion, the movement of the joint is more like a rhythmic movement rather than a stroke-based. Hence, Von-Mises basis functions *b*_*i*_ (Spiegelhalter et al., 2002) is used to model periodicity in the phase variable *z*,


(4) $$\begin{array}{l} b_{i}^{\text{‏}}\left(z_{t}\right)=\exp\left(\frac{\cos\left(2\pi \left(z_{t}-c_{i}\right)\right)}{h}\right), \end{array}$$

where *h* denotes the width of the basis and *c*_*i*_ is the center of the *i*th basis function. Then it is normalized by


(5) $$\begin{array}{l} \phi_{i}^{\text{‏}}\left(z_{t}\right)=\frac{b_{i}\left(z_{t}\right)}{\sum_{j=1}^{N}b_{j}\left(z_{t}\right)}. \end{array}$$

Then based on Paraschos et al. (2013) the probability of observing a trajectory *y*_*t*_ is introduced as,


(6) $$\begin{array}{l} p\left(y_{t}|\omega \right)=\prod_{t=1}^{T}N\left(y_{t}|\Phi_{t}\omega ,\sum_{y}\right). \end{array}$$

The probability distribution equation (6) depends on the parameter vector ω. Therefore, vector ω is the essential parameter for describing the trajectory, and what we mainly working on in this paper. In specific, according to Paraschos et al. (2013), a Gaussian distribution is assumed $p\left(\omega \;;\theta \right)=N\left(\omega |\mu_{\omega },\sum_{\omega }\right)$ with parameters θ to capture the variance of the trajectories by learning it. θ = {μ_ω_, ∑_ω_} is a set of parameters that specifies the mean and the variance of ω, which capture the similarities and differences of different realizations of the MPs.

To generate more reasonable motion, *p*(ω ; θ) need to be learned from multiple demonstrations. Assuming there are *M* demonstration trajectories, *M* sets of weight vectors can be obtained by linear fitting of the basis function. In this case, the weights for each trajectory are estimated using linear ridge regression,


(7) $$\begin{array}{l} \omega_{m}={\left(\Phi_{\text{‏}}^{{\text{‏}}^{T}}\Phi +\lambda I\right)}^{-1}\Phi_{\text{‏}}^{{\text{‏}}^{T}}Y_{m}. \end{array}$$

where *Y*_*m*_ represents the position of all steps for the *m*th demonstration trajectory, and λ = 0.1. Then the parameters θ = {μ_ω_, ∑_ω_} are obtained by the maximum likelihood estimation algorithm. The mean μ_ω_ and covariance ∑_ω_ are computed from samples ω_*m*_,


(8) $$\left\{ \begin{array}{l} \mu_{\omega }=\frac{1}{M}\sum_{m=1}^{M}\omega_{m}\\ \sum_{\omega }=\frac{1}{M-1}\sum_{m=1}^{M}\left(\omega_{m}-\mu_{\omega }\right){\left(\omega_{m}-\mu_{\omega }\right)}^{\text{T}} \end{array}\right.$$

Now the trajectory distribution *p*(*y*_*t*_ ; θ) can be defined by the hierarchical Bayesian model whose parameters are given by the parameters θ and the observation noise variance ∑_*y*_,


(9) $$\begin{array}{l} p\left(y_{t}\;;\theta \right)=\int p\left(\tau \;|\;\omega \right)p\left(\omega \;;\theta \right)d\omega \\ \;=\int N\left(y_{t}\;|\;\Phi_{t}\omega ,\Sigma_{y}\right)N\left(\omega \;|\;\mu_{\omega },\;\Sigma_{\omega }\right)d\omega \\ \;=N\left(y_{t}\;|\;\Phi_{t}\mu_{\omega },\;\Phi_{t}\sum_{\omega }\Phi_{t}^{T}+\Sigma_{y}\right). \end{array}$$

### Motion model compensation

MPs is a well-established approach for representing modular robot movement generators, due to their compact representation of the inherently continuous and high-dimensional robot movements. However, the wearable lower limb exoskeleton is a typical human-in-loop human-robot coupled system, so we should adapt ProMPs model to our local conditions by means of combining HRI with it. This combination can cooperate the human and exoskeleton together. Besides, HRI force is the most intuitive and practical way to estimate the user's intention, so the user's intentions are considered when generating trajectories. In this paper, the interaction between the user and the exoskeleton has been modeled, and then innovatively incorporated into the ProMPs as a compensation term. Then the trajectory generation for the current step can be affected by the HRI from the last step. Therefore, the linear basis function model of the trajectory becomes,


(10) $$\begin{array}{l} y_{{\text{‏}}_{t}}^{\text{I}}=\left[\begin{array}{c} q_{t}\\ {\dot{q}}_{t} \end{array}\right]=\Phi_{t}^{T}\omega +{\text{Ψ}}_{t}^{T}\omega_{\text{I}}+\varepsilon_{y} \end{array}$$

Where ${\text{Ψ}}_{t}=\left[\varphi_{t},{\dot{\varphi }}_{t}\right]$ is an *N* × 2 dimensional time-dependent basis function matrix. Gaussian function is adopted in here.

The weight vector ω_I_ is associated with the interaction force τ_I_ on each joint, then the trajectory generation will be affected by the real-time HRI force. In order to compensate for the track position more reasonably, discretize the interaction force into a form corresponding to ω_I_ by zero-order holder (ZOH)


(11) $$\begin{array}{l} \tau_{\text{I}}^{\text{D}}=\tau_{\text{I}}\left(1\left(t\right)-1\left(t{-}^{T_{\text{L}}}{/}_{N}\right)\right) \end{array}$$

Where 1(·) is a unit step function, and *T*_L_ is the period of the last gait step. There are total *N* force values of $F_{\text{I}}^{\text{D}}$ that are arranged in order. These values are denoted as a vector $F_{\text{I}}^{\text{D}}$. Now, we can obtain the weight vector ω_I_ as follows,


(12) $$\begin{array}{l} \omega_{\text{I}}=\alpha_{\text{I}}\tau_{\text{I}}^{\text{D}} \end{array}$$

Where α_I_ is the scaling factor.

### Motion model adaptive learning

Even though the trajectory can be compensated in real-time with HRI force, it still lacks agility when the system faces very different gait patterns. The exoskeleton is required to continuously improve the trajectory generation by optimizing the objective function. Hence, the decisive parameters ω in the motion generation model need to be updated constantly.

**Figure 2:**
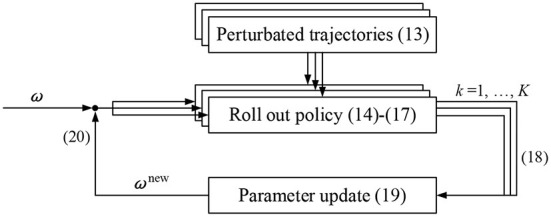
PI^BB^ policy improvement process. (13)-(20) Are corresponding formulas.

Figure 2 is the PI^BB^ motion learning policy improvement process. One execution of a policy is called a ‘roll-out'. In each iteration, the policy is perturbed and executed *K* times. A total of *K* alternative trajectories with slightly different is randomly generated around the last optimal trajectory. Based on these trajectories, policy improvement methods then update the parameter vector ω → ω^new^ such that the policy is expected to incur lower costs.

First, the policy parameter perturbation during a roll-out is generated from the model of the trajectory with noise


(13) $$\begin{array}{l} y_{t}^{{\text{‏}}^{\prime }}=\Phi_{t}^{T}\left(\omega +\varepsilon \right)\text{+}{\text{Ψ}}_{t}^{T}\omega_{\text{I}} \end{array}$$

Where ε is interpreted as a constant exploration noise.

The immediate cost function is the mismatch of the time point positions of trajectories,


(14) $$\begin{array}{l} r_{t}={\left(q_{t}^{{\text{‏}}^{\prime }}-q_{t}^{d}\right)}^{2} \end{array}$$

where $q_{t}^{d}$ represent the joint angle and the actual position of the last gait step. The trajectory cost *R* is,


(15) $$\begin{array}{l} R=\sqrt{\frac{1}{E}\sum_{t=1}^{E}r}. \end{array}$$

The cost function formula of each of the *k*th roll-out trajectory is computed with noise,


(16) $$\begin{array}{l} M_{t,k}=\frac{R^{-1}\Phi_{t,k}\Phi_{t,k}^{\text{T}}}{\Phi_{t,k}^{\text{T}}R^{-1}\Phi_{t,k}}, \end{array}$$

(17) $$\begin{array}{l} S_{k}=\phi_{E,k}+\sum_{t=0}^{E-1}r_{t,k}+\frac{1}{2}\sum_{t=1}^{E-1}{\left(\omega +M_{t,k}\varepsilon_{k}\right)}^{\text{T}}, \end{array}$$

where *M*_*t, k*_ is a projection matrix onto the range space of Φ_*t*_ under the metric *R*^−1^. ϕ_*E,k*_ is the terminal cost of *k*th trajectory, and *r*_*t,k*_ is the immediate cost of *k*th trajectory at time *t*.

The probability of the *k*th roll-out trajectory is obtained by mapping the cost of each trajectory to [0,1] through the Softmax function, as shown in equation (18),


(18) $$\begin{array}{l} P_{k}=\frac{e^{-\frac{1}{\gamma }S_{k}}}{\sum_{k=1}^{K}\left[e^{-\frac{1}{\gamma }S_{k}}\right]}, \end{array}$$

where the parameter γ is a constant coefficient within (0, 1]. It can be seen from equation (18) that the higher the cost, the lower the probability, thus ensuring PI^BB^ converges to the value with low cost.

For PI^BB^, the parameters are updated based on the scalar aggregated cost. Therefore, the parameter updating through reward-weighted averaging is,


(19) $$\begin{array}{l} \delta \omega =\sum_{k=1}^{K}\left[P_{k}\varepsilon_{k}\right]. \end{array}$$

The final parameter updates with,


(20) $$\begin{array}{l} \omega^{\text{new}}=\omega +\delta \omega . \end{array}$$

There are many index notations in this paper, so for the convenience of the readers, they are concluded: *i*th represents *N* basis functions; The *t*th of *E* number of time steps; The *k*th of *K* roll-out trajectories; The *m*th of *M* demonstration trajectories.

### Low-level control

The hierarchical control scheme needs a compliance control to work with motion learning results as a low-level control layer. The impedance control strategy emphasizes the active compliance of the exoskeleton system by establishing the dynamic relationship between the interaction force and the position. It can provide the exoskeleton with certain compliance while following the generated trajectory, and also allow the user to actively deviate from the desired trajectory to his comfortable way. In the application, the desired force τ_d_ is generated according to the position difference and the desired impedance model, then the desired force added to the compensation force τ_c_ calculated by the robot dynamics model is the joint driving force τ_r_, as shown in Figure 1 lower-level control. In this way, the robot system exhibits the desired characteristics of the impedance model. As for the mathematical description, a typical formulation of the impedance model is


(21) $$\begin{array}{l} \tau_{\text{d}}=M\left({\ddot{q}}_{\text{d}}-\ddot{q}\right)+B\left({\dot{q}}_{\text{d}}-\dot{q}\right)+K\left(q_{\text{d}}-q\right) \end{array}$$

Where *M* is the target impedance inertia parameter matrix; *B* is the damping, and *K* is the stiffness. ${\ddot{q}}_{d},{\dot{q}}_{d},q_{d}$ are the desired acceleration, velocity, and position of the exoskeleton, andare the corresponding actual values.

It can be seen from the formula that the choice of parameters directly determines the quality of the system control effect. The target impedance inertia parameter matrix *M* reflects the smoothness of the system response. *B* can reflect the energy consumed by the system. *K* measures the contact stiffness of the robot with the external environment. In our case, *M* is 1, *B* is 5 and *K* is 10.

As mentioned before, the dynamic model of the exoskeleton is required for this kind of impedance control. There are many ways to analyze robot dynamics. In this paper, the Lagrange equation is adapted which is standardized,


(22) $$\begin{array}{l} \tau_{\text{c}}=H\left(q\right)\ddot{q}+C\left(q,\ddot{q}\right)\dot{q}+G\left(q\right)\text{+}\tau_{f} \end{array}$$

Where *H*(*q*) is the Inertia matrix, $C\left(q,\ddot{q}\right)$ is the Centrifugal force and the Coriolis force matrix, *G(q)* is about gravity. τ_*f*_ is the friction.

The human gait dynamic model is complex, and the dynamic model varies with different gait phases. For the swing phase and standing phase, the models are simplified as two connecting rods and three connecting rods respectively. The process of the calculation and identification are not exhibited here in detail.

## Experiments and analyses

### Hardware

**Figure 3:**
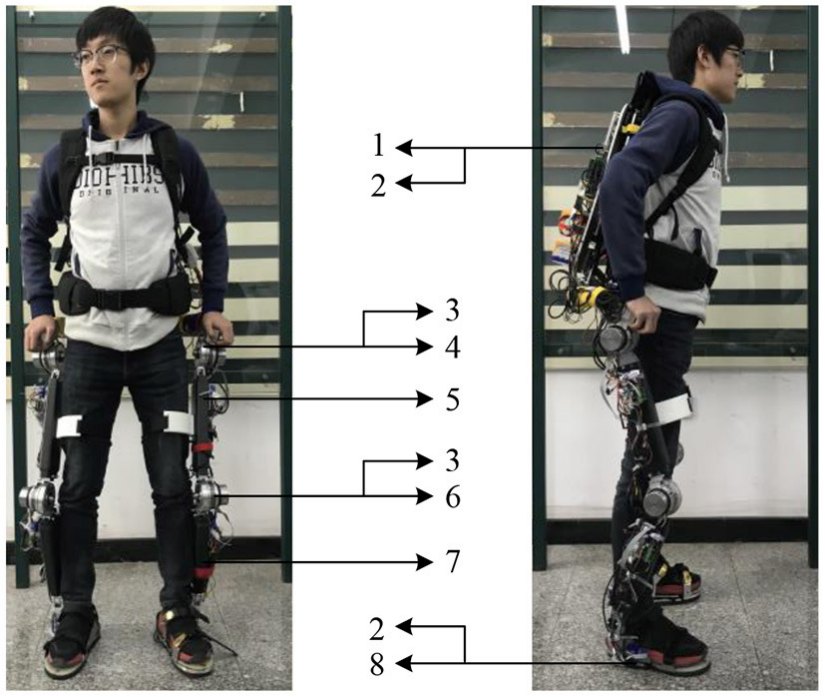
The hardware system of HEXO exoskeleton. 1. Backpack; 2. SFS; 3. Encode and torque sensor; 4. Hip joint; 5. IMU; 6. Thigh limb; 7. Knee joint; 8. Calf limb.

To verify the control scheme, real-time implementations were performed on an exoskeleton system named HEXO. Figure 3 shows the main components of the HEXO (Wang et al., 2022). The backpack is equipped with the ARM control board (ARM-Cortex-A9, ARM, UK), the power supply, and the data acquisition card. The lower limb exoskeleton is designed as an anthropomorphic device, so it has the same DOFs as the human lower limb. There are seven DOFs of the exoskeleton in total, four of which are active DOF (hip and knee flexion/extension DOFs). The actuation system is powered by a brushless DC motor (EC 60 flat, Maxon Motor, Switzerland), which is efficient and reliable. The incremental encoder (MILE Encoder 1024 CPT, Maxon Motor, Switzerland) is integrated into the motor. The servo drivers of these motors are arranged on the thigh segment and the shank segment respectively (G-SOLTWI 10/100SE, ELMO, Israel). The lower limb motion is measured by the inertial measurement unit (IMU) (LPMS-CU2, ALUBI, China) mounted on the thighs and shank carbon-fiber limbs to avoid the signal burrs caused by the angular difference. The torque sensors (type: 2210C, SRI, China) are placed at hip and knee joints, which are used to measure the torque of the joint. Besides, three six-axis force sensors (SFS) (M3715D, SRI, China) are installed at the back and sensing shoes between the user and the exoskeleton to perceive the human–robot interaction force. The sensing shoes also have four load cells (AT8106, AUTODA, China) for each shoe. All sensor data is transmitted to ARM (type: ARM-Cortex-A9, ARM, UK) through the CAN (Controller Area Network) bus, whose transmission rate is up to 1Mbits.

### Experimental protocol

**Figure 4:**
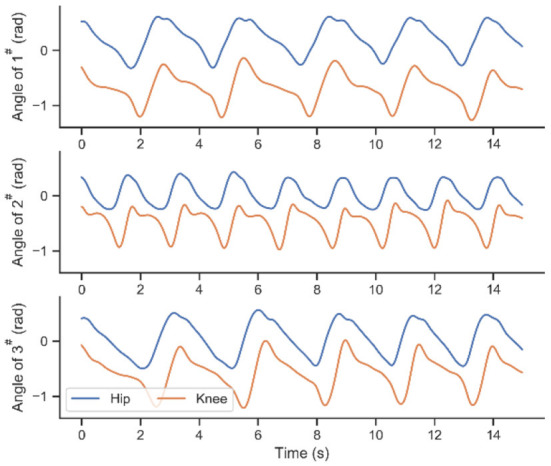
The walking trajectory data of subjects 1^#^, 2^#^, 3^#^.

Six healthy subjects (average height: 1.77 ± 0.07 m; averaged weight: 67.7 ± 10.1 kg) volunteered to participate in the experimental activities. As shown in Figure 1, in the process of adaptative motion learning, there is an initial modeling procedure need to be done before online application. A fundamental parameter set of ProMPs will be acquired in this offline procedure, and then the parameters will be updated in real-time. The walking data for the network modeling are collected from subjects 1^#^, 2^#^, and 3^#^ by performing the defined track under the zero-torque mode of the exoskeleton at a self-selected pace. Figure 4 exhibited part of the walking data profiles. As can be seen, the differences between each step are inevitable in walking even though participants are asked to keep their cadences constant during the execution of their track.

For online experiments, subjects 4^#^, 5^#^, and 6^#^ are recrewed. In order to testify the effectiveness of the proposed method, comparative experiments with different strategies are set up. Subjects 4^#^, 5^#^, and 6^#^ are asked to perform and repeat the track under different assistive-mode utilization at a self-selected pace. In both offline and online procedures, the system works at 100Hz. The four joints of the HEXO are processed simultaneously. All angle profile figures in this paper are from the right leg.

### Initial modeling

**Figure 5:**
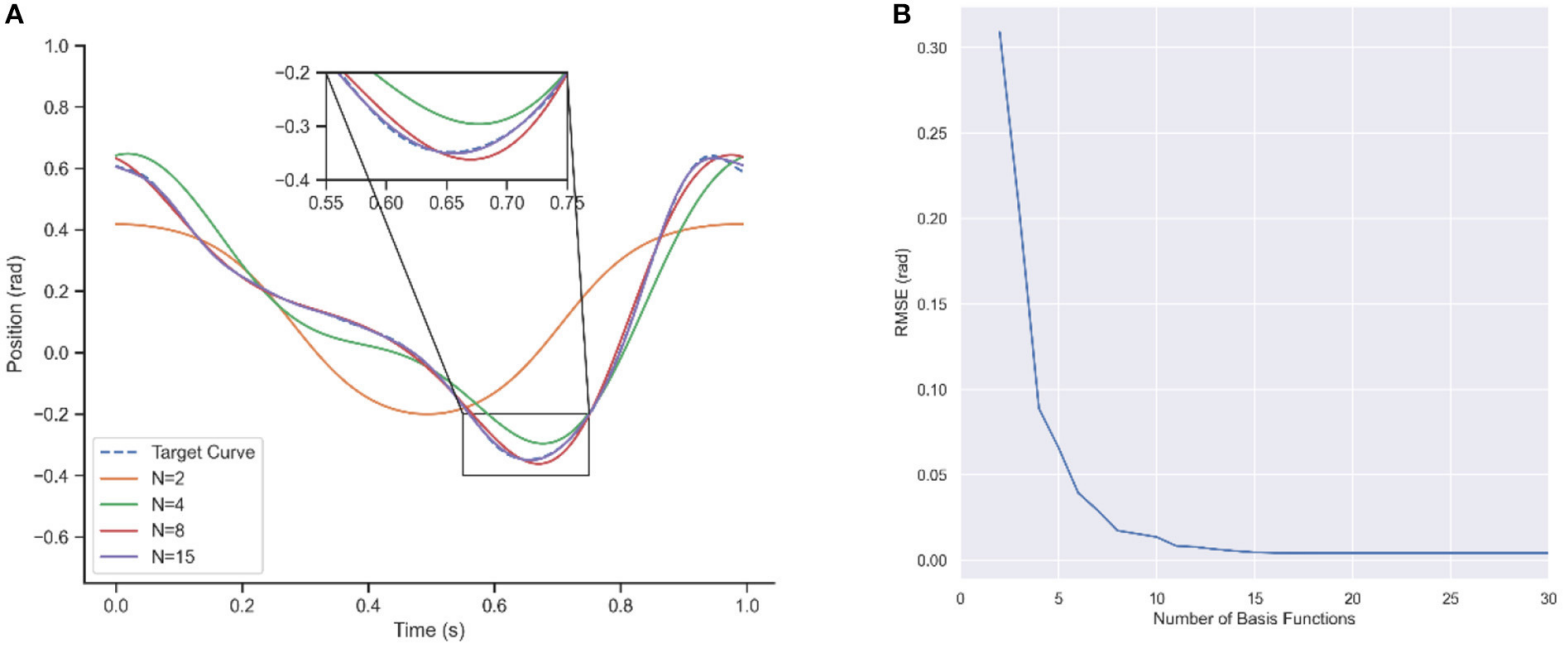
Trajectory learning results for different number of basis functions. **(A)** Curve after Learning. **(B)** RMSE of learning.

After trajectory data are obtained, the first step is to represent and model the trajectory by ProMPs. The process of ProMPs modeling the reference trajectory is described in section Motion generation model. The regression parameter λ in equation (8) is generally set to 10^-12^, and the basic function width *h* in equation (4) adopts 0.05. The number of basis functions *N* is a crucial factor in the representative ability of primitives. Thus, one step of the walking trajectory is learned firstly for choosing an appropriate *N*. Figure 5A shows the learning result under different *N*, and Figure 5B is the RMSE (Root Mean Square Error) between the learned trajectory and target trajectory. It can be seen that the representation ability is weak when the *N* is small which is normal, but it grows extremely faster with the increase of *N* compare to other primitives. The shape of the trajectory can be approximately described by 8 basis functions with 0.017 RMSE, and converged after 15 basis functions within 0.0046 RMSE. The trajectory learned by 10 basis functions which is the purple line in Figure 5A completely coincides with the target dotted blue one. Therefore, the basis function of ProMPs is adopted 15.

**Figure 6:**
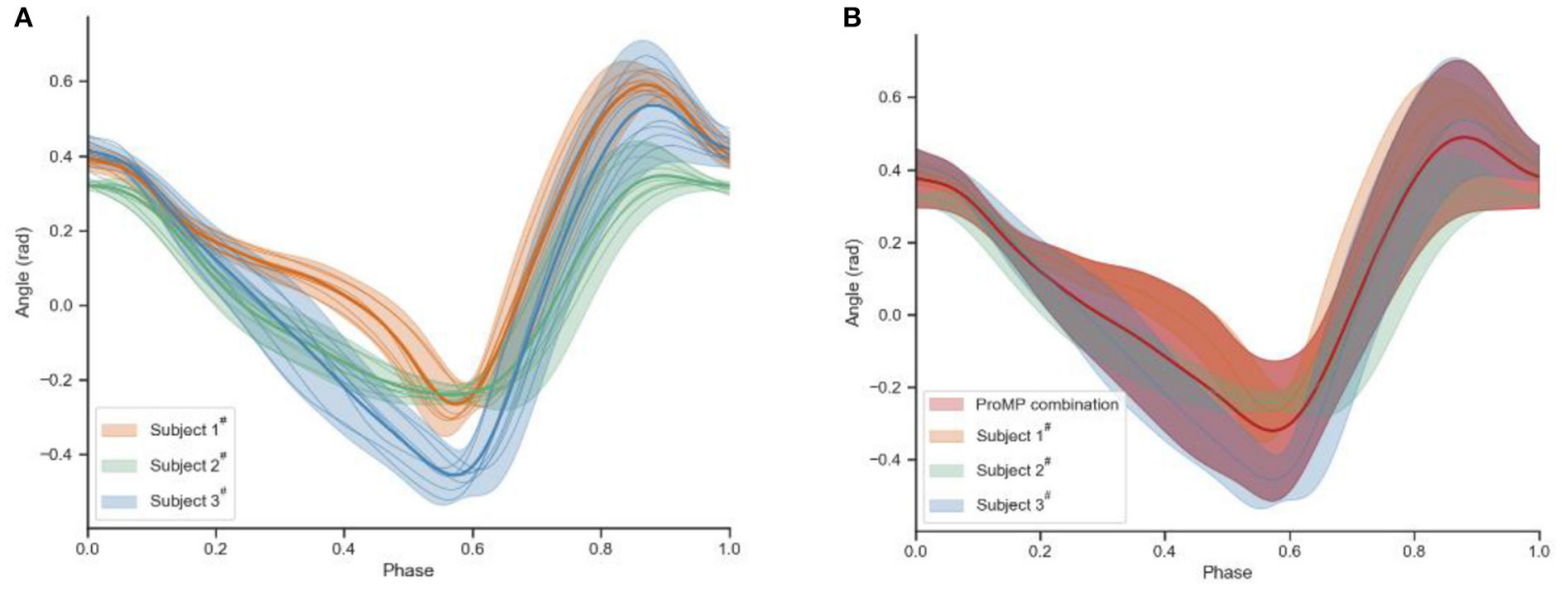
**(A)** The cut and normalized walking data of subjects 1#, 2#, 3#. **(B)** ProMPs learning result.

Then the walking data was fed to the ProMPs that were collected from three subjects 1#, 2#, and 3# in the formal initial modeling. Figure 6A shows the mean and covariance of the walking data of three subjects' right hip, which is cut and normalized based on the gait cycle. Only the profiles of the right hip joint are shown in this paper in order to be brief. The red area of Figure 6B is the result learned by ProMPs from all three of them, which contains all their possibilities. The red line can be regarded as the average of all acquired trajectories, so it is more representative of the characteristics of human gait behavior than any other ones. Besides, the more trajectories are learned, the more general the reference trajectory is.

### Experiments

For online experiments, the hardware platform is HEXO as mentioned before, embedding the corresponding control frame. The trajectory learned in section Initial modeling is regarded as the reference trajectory at the beginning. In order to test and verify the performance of the proposed method, we also set up other two baselines as comparisons. The first strategy denoted as “P-I” is the exoskeleton works only with the ProMPs model as motion learning. The model is unchangeable so the desired trajectory remains the same. The impedance control provides adequate compliance which makes sure the subjects can walk naturally in the way that he wants. In the second comparison “PP-I”, the motion learning model ProMPs can be constantly updated by the PI^BB^. The data from the actual joint trajectories are imported into the PI^BB^ algorithm to calculate the corresponding cost value, and the PI^BB^ adjusts the decisive parameters ω in motion learning according to the cost. Then a new trajectory with new characteristics is able to be generated. The last is the proposed interaction learning control strategy, entitle “PIP-I”. There is a dynamical interaction compensation term added into the motion learning part compared to the second “PP-I”, which is promising to reduce human–robot interaction force timely. Except that, the updating process is the same.

**Figure 7:**
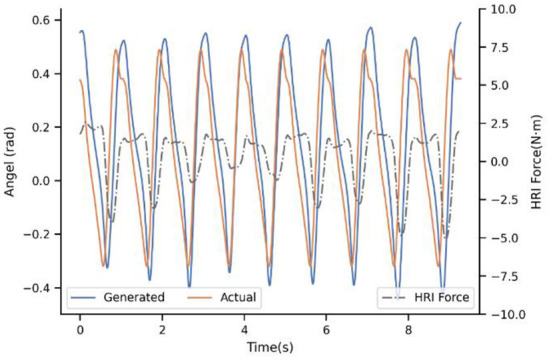
Experimental results of subject 4^#^ with strategy P-I.

Figure 7 shows the result of the first baseline P-I. It compares the generated trajectory and the actual one at the beginning of the walking of subject 4^#^. Note that the actual trajectory represents the intention of the users' in this situation. In practice, the reference trajectories learned by ProMPs still differ across the users, maybe owing to the learning samples of the ProMPs model is not enough based on our limited experimental conditions. However, even though there are much more samples to learn, the difference cannot be eliminated due to different physical characteristics and the uniqueness of the human gait. In addition, it can be seen that the generated trajectory is the same for every step if there is no adaptation of the model, and when the step time between the actual and generated one are unsynchronized then the generated angel keeps the last value. Usually, the impedance control will drive the actual angle close to the ideal to a certain extent, but we ask subjects to insist on their own walking way because the ultimate goal is exoskeleton actively consistent with the humans instead of the opposite. Therefore, there are mismatches between the desired trajectory and the actual one. In this way, it will cost a lot to maintain the gait of the subjects, and the HRI is not ideal without a doubt. The HRI force is an intuitive benchmark to measure the performance of synchronization.

**Figure 8:**
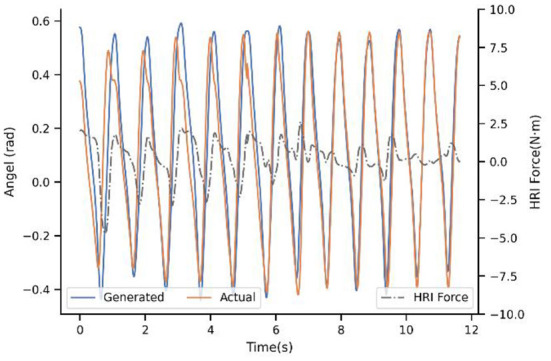
Experimental result of subject 4^#^ with strategy PP-I.

With regard to the second strategy PP-I, the experimental result is demonstrated in the Figure 8. The updating rate is one step, which is also the minimal unit because of the motion trajectory that ProMPs modeled is for every step. For the first step, there still is a big difference. Nevertheless, with the help of the adaptation, the generated trajectory is adjusting constantly by the PI^BB^, and closely follows the actual after 8-9 steps. Moreover, the HRI force decreased a lot after convergence, as a result of trajectory adaptation which decreased the conflict between human and exoskeleton. That is to say, PP-I is able to provide a more coordinated HRI for the system and a more comfort assistant experience.

**Figure 9:**
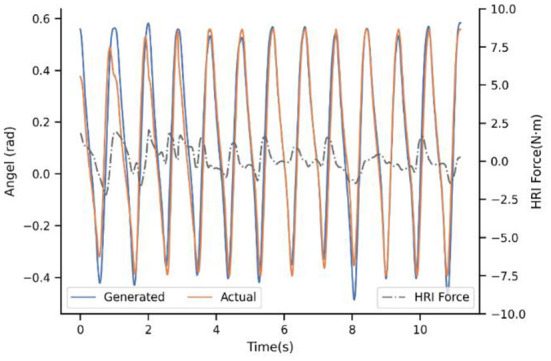
Experimental result of subject 4^#^ with strategy PIP-I.

Up to now, the superiority of dynamic adaptation is quite apparent. Adjusting the generated trajectory constantly adds flexibility to the system, by enhancing the ability to adapt to the new situation. However, the adaptation rule is only extracted from trajectory mismatch seems inadequate, which brought the next experiment. Figure 9 illustrates the adaption performance of PIP-I, which is the proposed method in this paper. It indicates that the convergence trend is similar to PP-I, but the convergence speed is improved a lot. For subject 4^#^, the convergence only takes 3-5 steps. The underlying reason is that our adaptation is extracted from a cost function that penalizes both interaction and trajectory mismatch, by adding the HRI compensation term into the motion generation model.

**Figure 10:**
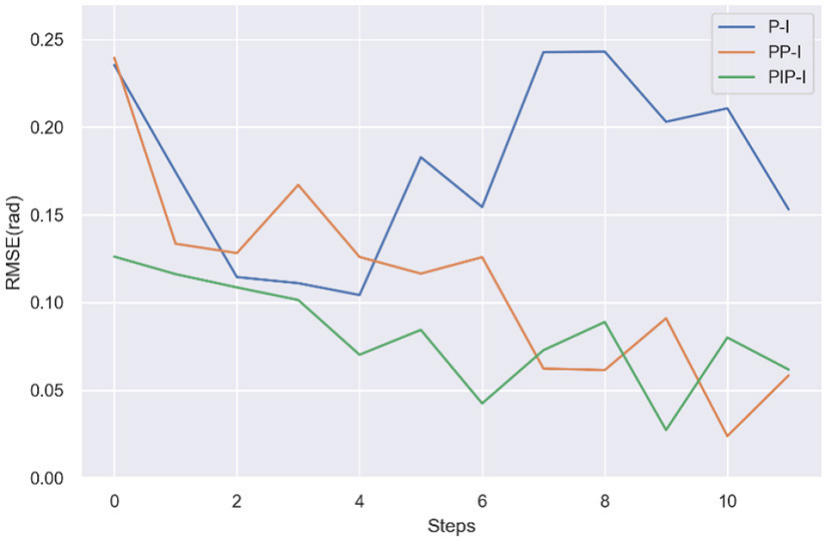
Comparison of the RMSE of the mismatch error of three strategies, P-I, PP-I, and PIP-I.

Figure 10 compares the average mismatch errors of three strategies. Note that, comparing the errors of the first step is of no necessity because the value is random. After the first step, the errors gradually reduced both in PP-I and PIP-I, while there is no obvious convergence in P-I. There is not much difference in steady state error of PP-I and PIP-I, but the PIP-I approaches the stable error faster. The decrease in mismatch errors indicates that adapted trajectories are more consistent with the human-exoskeleton dynamics. It is a very promising result for the proposed method, in terms of the exoskeleton system can consistent with the different users more quickly.

**Figure 11:**
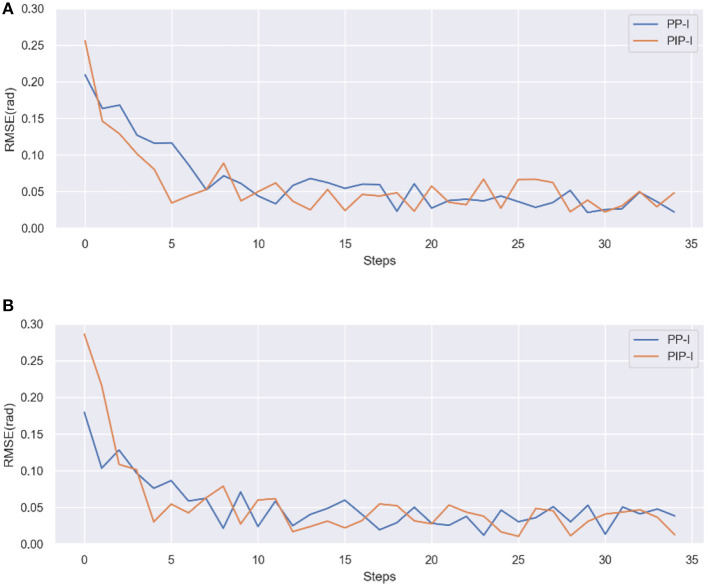
The performances of PP-I and PIP-I in RMSE of error profiles. **(A)** Subject 5^#^. **(B)** Subject 6^#^.

Figure 11 illustrates the experimental result of error profiles on subjects 5^#^, 6^#^. The results of baseline P-I are the same as the subject 4^#^, so it will not be pointed out in further detail. For the PP-I and PIP-I, PIP-I shows the absolute advantage of convergence speed in all cases. It is hard to tell which step the errors converges to, but it is about 7-10 steps for PP-I while only 3-6 steps are needed by PIP-I for all three subjects.

The interaction force was almost proportional to the errors and followed the same trend as the errors. In Table 1, we show the average HRI force of the 4–6th step and 9–11th step respectively. We can see from the Table 1, that the HRI force with P-I is not changed as the number of steps increases. PP-I reduces the force to stable until the 9–11th step, but PIP-I can reach the steady states just with only the 4–6th steps needed. Although there is the effect of the gait randomicity, it is still can be drawn that PIP-I is able to reduce the adaptation time nearly by half. As a matter of fact, adjustment time halved has a great impact on the actual wearing experience. Moreover, according to the subjects, it is stable and comfortable when the exoskeleton works on PP-I and PIP-I, and the adaption process of PIP-I is rapid and hardly conscious. Consequently, the experiments exhibited that the proposed method can model the human trajectory and perceive the human intention online with not only low error but also, most importantly, high efficiency. With this dynamical interaction learning control, the lower limb exoskeleton can provide natural and comfortable assistance to the users in the way that he wants, and the system can also adapt to different users and situations due to the adaptive updating procedure. With the HRI compensation term, the flexibility and coordination of the human-robot system are further improved.

**Table 1:** The comparison of HRI force performance for strategies P-I, PP-I, and PIP-I.

**Average HRI force(N m)**	**4–6th step**			**9–11th step**		
	**P-I**	**PP-I**	**PIP-I**	**P-I**	**PP-I**	**PIP-I**
Subject 4^#^	1.21	1.20	0.74	2.09	0.48	0.56
Subject 5^#^	2.65	1.10	0.62	2.12	0.49	0.47
Subject 6^#^	1.86	0.78	0.55	1.93	0.28	0.44

## Conclusion and future work

We presented a novel interaction learning control framework for the lower limb exoskeleton to naturally assist people. The motion learning model generates desired trajectory online which is a combination of movement primitives and human–robot interaction force, and it is adjustable to converge to human intention and adapt to different users. We firstly adopted ProMPs to model the human motion trajectory in the lower limb exoskeleton, and in this paper, it is further integrated with HRI working on assistive mode. Furthermore, the motion learning model is constantly updated online by PI^BB^, which can ensure the adaptability of the method to different gait patterns of various users. The experiments reveal that the proposed strategy can timely provide a smooth and natural trajectory online which is in line with the user's pattern so that the exoskeleton system could cooperate with the human user with smaller HRI. Most importantly, the convergence time is further reduced by adding the HRI compensation term, which improved the efficiency of the system and its comfort. Our analysis and experiment results show the applicability and effectiveness of the proposed method and its feasibility to be used in lower limb exoskeletons.

For this paper, the locomotion mode involved in testing is only level walking. In the future, all basic rhythmic locomotion modes in daily living will be included, such as stair ascent, stair descent, ramp ascent, and ramp descent. The performance of the learning should be similar since there is no essential difference between these motions. Besides, the situation of speed changing in walking should be taken into consideration, so the adaptability of the method for that need to be verified further.

## Data availability statement

The original contributions presented in the study are included in the article/supplementary material, further inquiries can be directed to the corresponding author/s.

## Ethics statement

The studies involving human participants were reviewed and approved by Heilongjiang Provincial Hospital. The patients/participants provided their written informed consent to participate in this study. Written informed consent was obtained from the individual(s) for the publication of any potentially identifiable images or data included in this article.

## Author contributions

JW proposed the methods and conducted the experiments and wrote the manuscript. DW, YG, and WD supervised the whole process. All authors contributed to the article and approved the submitted version.
